# Association between Geriatric Nutrition Risk Index and depression in older hemodialysis patients with and without type 2 diabetes mellitus: a multicenter cross-sectional study

**DOI:** 10.3389/fendo.2025.1646514

**Published:** 2025-09-29

**Authors:** Jinwen Zhang, Jing Gao, Siqin Gaowa, Peipei Han, Xiaoyu Chen, Pingping Cai, Jiangling Guo, Qianhao Wu, Jingjie Miao, Chengzhang Zhao, Keying Zhang, Lingyao Kong, Jing Shui, Qi Guo

**Affiliations:** ^1^ Shanghai University of Traditional Chinese Medicine, Shanghai, China; ^2^ Department of Rehabilitation Medicine, Shanghai University of Medicine and Health Sciences, Shanghai, China; ^3^ General Practice Clinic, Pujiang Community Health Service Center in Minhang District, Shanghai, China; ^4^ Department of Cardiovascular, Inner Mongolia People’s Hospital, Inner Mongolia, China; ^5^ School of Health, Fujian Medical University, Fujian, China

**Keywords:** Geriatric nutritional risk index, depression, type 2 diabetes mellitus, hemodialysis, malnutrition

## Abstract

**Objectives:**

The purpose of this study was to observe the relationship between the Geriatric nutrition risk index (GNRI) and depression in the presence or absence of type 2 diabetes mellitus (T2DM) in older adults undergoing hemodialysis.

**Design:**

In this multicenter cross-sectional study, 684 clinically stable hemodialysis patients aged ≥60 years (431 men; mean age: 69.6 ± 6.6 years) were included from seven dialysis units in Shanghai, China. *Measures*: Depressive symptoms were assessed using the PHQ-9 scale, and T2DM diagnoses were determined by medical records. Multinomial logistic regression was performed to evaluate the association between Geriatric Nutritional Risk Index (GNRI) and depression.

**Results:**

Hemodialysis patients with diabetes had a high prevalence of depression (39.4%). In diabetes patients, GNRI was associated with depression after adjusting covariates [OR=0.984; 95% confidence interval (CI) = 0.969–0.999, *P*=0.046]. However, no significant association was found between GNRI and depression in the non-diabetes hemodialysis patients (*P* > 0.05).

**Conclusions:**

This cross-sectional study examines the relationship between the GNRI and depression in hemodialysis patients with T2DM rather than the non-T2DM group. Further studies are needed to investigate more causal relationships between GNRI and depression in patients with T2DM.

## 1 Introduction

End-stage renal disease (ESRD) is a significant global public health concern that affects people in both wealthy and developing nations (1). Renal replacement therapy is currently provided to more than 1.9 million patients with ESRD worldwide (2). Patients on hemodialysis (HD) encounter a variety of health-related stressors, significantly increasing their susceptibility to depression (3). Of particular concern is the high prevalence of depression among patients receiving HD. A recent study reported that 74.6% of patients on HD had clinically significant depressive symptoms (4). Among patients with HD, depression adversely affects clinical outcomes, manifesting as diminished quality of life, compromised medication adherence, heightened hospital readmission rates, and elevated risks of both suicidal behavior and mortality (5–7).

Type 2 diabetes mellitus (T2DM) is characterized by chronic hyperglycemia, while depression is a prevalent comorbidity of the condition, probably as a result of overlapping risk factors (8). Studies have indicated that individuals with diabetes have a higher prevalence of depression (9). Biological pathways, including chronic hyperglycemia, low-grade inflammation, and microvascular dysfunction, are implicated in the pathogenesis of both T2DM and depression (10). Subclinical inflammation, dysregulation of the hypothalamic–pituitary–adrenal (HPA) axis, and the sympathetic nervous system are linked to molecular alterations in depression and T2DM (11). Depression predicts suboptimal glycemic control, more frequent hospitalizations, accelerated diabetic complications, and increased diabetes-related mortality (12). Individuals with diabetes mellitus who exhibit poor glycemic control are at an increased risk of incident depression and other associated health complications (13). Depression can significantly impact daily behaviors, including sleep patterns, dietary intake, appetite, fatigue levels, and physical activity. These symptoms are also closely associated with diabetes management. Patients diagnosed with comorbidities may derive clinical benefit from enhanced surveillance protocols and individualized therapeutic interventions (14–16).

Malnutrition represents a significant public health concern, particularly among elderly individuals residing in long-term care facilities, hospitalized patients, or those living independently at home (17–19). Previous research has demonstrated that 27.3% of HD patients exhibit moderate to severe malnutrition (20). The Geriatric Nutritional Risk Index (GNRI), introduced by Bouillanne et al. in 2005, is an objective nutritional screening tool based on height, weight, ideal body weight, and serum albumin concentration. This tool has been validated as a reliable instrument for assessing patients’ nutritional status and identifying individuals at risk of malnutrition (21, 22). Existing literature has established an association between dehydration and depression, with evidence indicating that malnutrition tends to be more pronounced among dialysis patients experiencing depressive symptoms (23). Malnutrition is also one of the most common complications in older people with T2DM. Studies on the connection between GNRI and depression in older adults with T2DM are limited, particularly in the settings of Chinese community dialysis patients.

The purpose of this study was to examine the association between GNRI and depression in older Chinese community-dwelling dialysis patients, stratified by the presence or absence of T2DM. Diabetes stratification is crucial for identifying and managing high-risk patients susceptible to complications, particularly within the dialysis population. Accurate risk stratification enables the customization of treatment plans, thereby enhancing the overall care provided to patients. We hypothesized that lower GNRI scores would be associated with an increased prevalence of depression among patients with T2DM on dialysis. By examining the relationship between depression and GNRI, dialysis patients can be provided with more personalized nutritional and psychological interventions, thereby enhancing their overall quality of life and treatment satisfaction.

## 2 Materials and methods

### 2.1 Study participants

The multicenter cross-sectional study recruited patients who underwent hemodialysis in seven dialysis units in Shanghai, China, between July 2020 and March 2023. Individuals were included if they met the following criteria: (1) older adults aged ≥60 years, (2) who had been on maintenance hemodialysis for at least 3 months, and (3) who were willing to participate in this study. Participants with the following conditions were excluded from the study: (1) unable to communicate with interviewers or to grant informed consent; (2) did not have complete information on depression, nutritional assessment, and T2DM; (3) those who did not have a blood sample taken; (4) those who were unable to complete a physical performance test. Following these exclusions, the final analyzed population comprised 684 subjects. The study was approved by the Ethics Committee of Shanghai University of Medicine and Health Sciences, and the methods were carried out in accordance with the principles of the Declaration of Helsinki. All participants were informed and signed consent prior to enrollment in the study.

### 2.2 Definition of depression

The PHQ-9 has shown significant screening efficacy in a variety of populations, including patients in Chinese primary care settings (24, 25). The PHQ-9 score consists of nine questions scored on a scale of 0–3 based on symptom frequency. Patients reported the frequency with which they had experienced the following nine symptoms of major depressive disorder: (1) anhedonia, (2) depressed mood, (3) sleep disturbance, (4) fatigue, (5) appetite changes, (6) low self-esteem, (7) concentration problems, (8) psychomotor disturbances, and (9) suicidal ideation. Total scores range from 0 to 27 (26). Patients were considered to be depressed if the PHQ-9 score was ≥5 (27–29). In this study, the Cronbach’s α coefficient for this scale was 0.886.

### 2.3 Assessment of nutritional status

GNRI is equal to [41.7 × (actual weight/ideal weight)] + [1.489 × serum albumin (g/L)]. Ideal and real body weights showed weight decrease. Different Lorenz formulas are used for determining optimal weight depending on gender. The optimal weight calculation for men is 0.75 × height (cm) - 62.5, whereas the ideal weight calculation for women is 0.60 × height (cm) - 40. The actual weight/ideal weight is set to one if the actual weight is higher than the ideal weight (30).

### 2.4 Type 2 diabetes mellitus assessment

Based on the participants’ self-reports, we were able to get diabetes information. We also double-checked the fasting plasma glucose (FPG) data by looking up the information in electronic medical records. The American Diabetes Association 2021 criteria defined diabetes as having an FPG level of at least 7.0 mmol/L, a 2h plasma glucose level of at least 11.1 mmol/L on an oral glucose tolerance test, or an HbA1c of at least 6.5% (31).

### 2.5 Covariates

Using standardized questionnaires and in-person interviews, information on health behaviors (such as alcohol use and smoking) and demographic traits (such as age, gender, and education) was gathered. We measured depression symptoms with the Patient Health Questionnaire (PHQ-9). The GNRI, was used to evaluate nutritional status. Over the course of three months, biochemical data were gathered, including serum albumin, hemoglobin, calcium, phosphorus, and parathyroid hormone (PTH).

### 2.6 Statistical analysis

Based on whether the patients’ baseline characteristics were consistent with depression, we divided the sample into two groups. In our study, a limited number of continuous variables exhibited missing values, all with a missing proportion <5%. For variables following a normal distribution, mean imputation was applied; for those deviating from normality, the corresponding median was used so as to minimize bias. Mean standard deviations are used to express continuous variables, medians and quartiles are used to express skewed continuous variables (like GNRI), and percentages are used to express categorical variables. Comparisons between two different groups (depression vs. non-depression and depression in T2DM vs. depression in non-T2DM) were made using the chi-square test for categorical variables, the Kruskal–Wallis test for skewed continuous variables, and the t-test for normally distributed continuous variables.

The relationships between GNRI and depression were investigated using logistic regression analysis, first for the entire sample and subsequently for each group. Depression was the outcome variable, which may be either 1 or 0. Regression models contained covariates that differed significantly between groups. These included age, sex, BMI, GNRI, smoking, alcohol use, hypertension, hyperlipidemia, vintage, heart disease, and medicine use, among other sociodemographic characteristics. The Statistical Package for the Social Sciences (SPSS) version 26.0 (IBM Corp., Armonk, New York) was used to analyze all the data, and *P <*0.05 was chosen as the significant level.

## 3 Results

**Figure 1:**
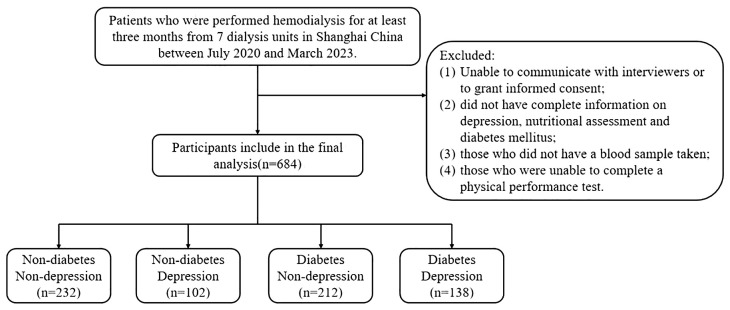
Flow diagram of the study.

**Figure 1** shows the flow of hemodialysis participants with subgroups. Baseline characteristics of the subjects were presented in **Table 1**. Among 684 participants (431 men, 253 women; mean age 69.60 ± 6.65 years), there were 240 (35.1%) patients with depression. **Table 1** shows the socioeconomic and health-related characteristics of patients with MHD stratified by depression. Hyperlipidemia, diabetes, heart disease, and PTH significantly differed between groups (*P*<0.05, **Table 1**).

**Table 1:** Baseline characteristics of study participants with and without depression.

Characteristics	Non-depression (*N*=444)	Depression (*N*=240)	*P*-value
Age(y)	69.53 ± 6.59	69.73 ± 6.77	0.708
Sex(*n*,%)			0.230
Male	287(64.6)	144(60.0)	
Female	157(35.4)	96(40.0)	
BMI(kg/m^2^)	23.40 ± 3.61	23.22 ± 3.42	0.528
Education(*n*,%)			0.321
Illiterate	35(7.9)	14(5.8)	
Non- Illiterate	409(92.1)	226(94.2)	
Alcohol use(*n*,%)			0.162
Yes	206(46.4)	98(40.8)	
No	238(53.6)	142(59.2)	
Smoke(*n*,%)			0.631
Yes	225(50.7)	117(48.8)	
No	219(49.3)	123(51.2)	
Creatinine index	19.94 ± 5.18	20.47 ± 2.61	0.078
Vintage (months)	36.83(17.18,72.13)	45.07(19.51,87.53)	0.081
GNRI(*n*,%)	99.49 ± 13.47	97.01 ± 17.53	0.528
Hypertension(*n*,%)			0.228
Yes	409(92.1)	227(94.6)	
No	35(7.9)	13(5.4)	
Hyperlipidemia(*n*,%)			0.024
Yes	129(29.1)	90(37.5)	
No	315(70.9)	150(62.5)	
Diabetes(*n*,%)			0.015
Yes	212(47.7)	138(57.5)	
No	232(52.3)	102(42.5)	
Heart disease(*n*,%)			0.015
Yes	123(27.7)	88(36.7)	
No	321(72.3)	152(63.3)	
Medicine use			0.177
0	16(3.6)	4(1.7)	
1 or 2	75(16.9)	33(13.8)	
≥3	353(79.5)	203(84.6)	
Laboratory parameters			
Hemoglobin(g/L)	110.90 ± 16.66	111.50 ± 14.69	0.638
Albumin(g/L)	38.63 ± 4.10	40.16 ± 19.40	0.110
Sodium(mmol/L)	137.89 ± 7.60	138.78 ± 2.58	0.077
Phosphor(mmol/L)	2.58 ± 14.35	1.85 ± 0.60	0.435
Calcium(mmol/L)	2.83 ± 11.53	2.27 ± 0.25	0.459
PTH (pg/ml)	277.32 ± 251.44	348.81 ± 288.64	0.001

BMI, body mass index; GNRI, Geriatric Nutritional Risk Index; PTH, parathyroid hormone.

Data are presented as mean ± SD or *n* (%).

**Figure 2:**
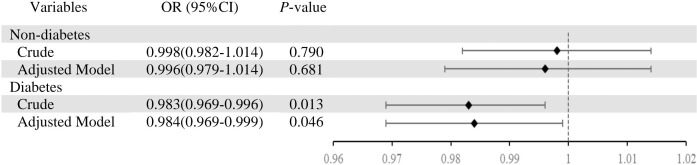
Association between depression (depression vs. non-depression) and geriatric nutritional risk index in total sample and by type 2 diabetes mellitus. Adjusted model was adjusted for age, sex, BMI, alcohol use, smoke, vintage, hypertension, hyperlipidemia, heart disease, medicine use.

In participants with T2DM, we classified them again according to whether they are defined as depressed (**Table 2**). Individuals with T2DM who concurrently had depression showed reduced GNRI scores (*P* =0.017). Individuals without T2DM who suffered from depression required lengthier dialysis sessions (*P* =0.005). Heart disease (*P* =0.004) and hyperlipidemia (*P* =0.018) were also more common in people with depression. Additionally, individuals with depression had higher levels of laboratory markers such as hemoglobin (*P* =0.037), albumin (*P* =0.049), and PTH (*P* =0.002). **Table 2** shows the difference in characteristics of older adults classified by depression and T2DM.As shown in **Figure 2**, it is noteworthy that in the diabetes group, depression patients’ GNRI scores was significantly associated with depression and it is a protective factor for depression.

**Table 2:** Baseline characteristics of subjects classified by T2DM and depression.

Characteristics	Non-T2DM (*n*=334)		*P*-value	T2DM (*n*=350)		*P*-value
	Non-depression (*n*=232)	Depression (*n*=102)		Non- depression (*n*=212)	Depression (*n*=138)	
Age(y)	70.08 ± 7.01	69.58 ± 6.42	0.539	68.93 ± 6.06^a^	69.84 ± 7.04^a^	0.213
Sex(*n*,%)			0.515			0.129
Male	134(57.8)	55(53.9)		153(72.2)^a,b^	89(64.5)	
Female	98(42.2)	47(46.1)		59(27.8) ^a,b^	49(35.5)	
BMI(kg/m^2^)	22.83 ± 3.49	22.88 ± 3.08^a^	0.896	24.03 ± 3.64^a,b^	23.47 ± 3.65	0.165
Education(*n*,%)			0.369			0.812
Illiterate	23(9.9)	7(6.9)		12(5.7)	7(5.1)	
Non- Illiterate	209(90.1)	95(93.1)		200(94.3)	131(94.9)	
Alcohol use(*n*,%)			0.306			0.217
Yes	98(42.2)	37(36.3)		108(50.9)	61(44.2)	
No	134(57.8)	65(63.7)		104(49.1)	77(55.8)	
Smoke(*n*,%)			0.704			0.470
Yes	103(44.4)	43(42.2)		122(57.5)^a^	74(53.6)	
No	129(55.6)	59(57.8)		90(42.5)^a^	64(46.4)	
Creatinine index	19.80 ± 5.35	20.59 ± 2.47	0.066	20.10 ± 5.00	20.38 ± 2.71	0.496
Vintage (months)	43.57(19.6,91.7)	66.3(32.9,124.2)	0.005	32.1(14.1,56.5)	36.2(14.3,58.4)	0.629
GNRI(score)	98.30 ± 13.58	97.85 ± 15.91	0.790	100.78 ± 13.27	96.39 ± 18.68^c^	0.017
Hypertension (*n*,%)			0.623			0.402
Yes	205(88.4)	92(90.2)		204(96.2)^a^	135(97.8)^a^	
No	27(11.6)	10(9.8)		8(3.8)^a^	3(2.2)^a^	
Hyperlipidemia (*n*,%)			0.018			0.564
Yes	51(22.0)	35(34.3)		78(36.8)^a^	55(39.9)^a^	
No	181(78.0)	67(65.7)		134(63.2)^a^	83(60.1)^a^	
Heart disease(*n*,%)			0.004			0.631
Yes	53(22.8)	39(38.2)^a^		70(33.00	49(35.5)	
No	179(77.2)	63(61.8)^a^		142(67.0)	89(64.5)	
Medicine use			0.213			0.404
0	10(4.3)	1(1.0)		6(2.8)	3(2.1)	
1 or 2	42(18.1)	18(17.6)		33(15.6)	15(10.9)	
≥ 3	180(77.6)	83(81.4)		173(81.6)	120(87.0)	
Laboratory parameters						
Hemoglobin(g/L)	111.4 ± 17.7	115.6 ± 14.5^a^	0.037	110.40 ± 15.49^b^	108.54 ± 14.21^b^	0.248
Albumin(g/L)	38.58 ± 4.39	42.19 ± 29.37^a^	0.049	38.68 ± 3.74^b^	38.67 ± 3.88^b^	0.934
Sodium(mmol/L)	138.26 ± 4.38	139.16 ± 2.33	0.052	137.48 ± 10.00	138.51 ± 2.73	0.240
Phosphor(mmol/L)	3.21 ± 19.84	1.90 ± 0.53	0.508	1.89 ± 0.59	1.82 ± 0.65	0.287
Calcium(mmol/L)	2.36 ± 1.60	2.30 ± 0.23	0.738	3.34 ± 16.61	2.26 ± 0.26	0.442
PTH (pg/ml)	294.28 ± 290.31	409.29 ± 342.55^a^	0.002	258.77 ± 199.50^b^	304.11 ± 232.60^b,c^	0.053

BMI, body mass index; GNRI, Geriatric Nutritional Risk Index; PTH, parathyroid hormone.

Data are presented as mean ± SD or *n* (%).

**^a^** *P <*0.05, compared with non-T2DM and non-depression group; **^b^** *P <*0.05, compared with non-T2DM and depression group; **^c^** *P <*0.05, compared with T2DM and non-depression group.

We further analyzed the relationship between depression and GNRI in T2DM participants (**Table 3**). Before adjustment for covariates, there were significant differences between baseline depression and GNRI (OR=0.983, 95% CI 0.969–0.996, *P* =0.013). After adjusting for covariates, significant differences between baseline depression and GNRI during follow-up (OR=0.984, 95% CI 0.969–1.000, *P* =0.046).

**Table 3:** Logistic regression analysis of the continuous Geriatric Nutritional Risk Index on the presence of depression in the non-diabetic and diabetic hemodialysis patients.

Characteristics	Non-T2DM				T2DM			
	Crude		Adjusted model		Crude		Adjusted model	
	OR (95% CI)	*P*	OR (95% CI)	*P*	OR (95% CI)	*P*	OR (95% CI)	*P*
GNRI(score)	0.998(0.982–1.014)	0.790	0.996(0.979–1.014)	0.681	0.983(0.969–0.996)	0.013	0.984(0.969–0.999)	0.046
Age(y)	0.989(0.956–1.024)	0.538	0.987(0.950–1.025)	0.498	1.022(0.989–1.056)	0.199	1.017(0.982–1.054)	0.343
Sex (*n*,%)								
Male	Ref.	–	Ref.	–	Ref.	–	Ref.	–
Female	1.168(0.731–1.867)	0.515	0.972(0.460–2.052)	0.940	1.428(0.901–2.262)	0.129	1.565(0.783–3.126)	0.205
BMI(kg/m^2^)	1.005(0.937–1.077)	0.895	1.017(0.937–1.103)	0.689	0.958(0.902–1.018)	0.166	0.979(0.914–1.049)	0.549
Alcohol use (*n*,%)								
No	Ref.	–	Ref.	–	Ref.	–	Ref.	–
Yes	1.285(0.795–2.077)	0.307	1.443(0.748–2.781)	0.274	1.311(0.852–2.016)	0.218	1.202(0.692–2.088)	0.514
Smoke (*n*,%)								
No	Ref.	–	Ref.	–	Ref.	–	Ref.	–
Yes	1.096(0.684–1.754)	0.704	1.157(0.564–2.376)	0.691	1.172(0.762–1.805)	0.470	0.805(0.407–1.592)	0.534
Vintage (months)	1.004(1.001–1.008)	0.015	1.005(1.001–1.009)	0.008	0.999(0.995–1.004)	0.786	0.999(0.993–1.004)	0.588
Hypertension (*n*,%)								
No	Ref.	–	Ref.	–	Ref.	–	Ref.	–
Yes	1.212(0.563–2.607)	0.623	1.338(0.581–3.083)	0.494	1.765(0.460–6.771)	0.408	1.555(0.378–6.405)	0.541
Hyperlipidemia (n,%)								
No	Ref.	–	Ref.	–	Ref.	–	Ref.	–
Yes	1.854(1.109–3.098)	0.018	1.691(0.972–2.944)	0.063	1.138(0.733–1.768)	0.564	1.153(0.720–1.845)	0.553
Heart disease(*n*,%)								
No	Ref.	–	Ref.	–	Ref.	–	Ref.	–
Yes	2.091(1.264–3.459)	0.004	2.490(1.440–4.303)	0.001	1.117(0.711–1.754)	0.631	0.981(0.607–1.586)	0.939
Medicine use								
0	Ref.	–	Ref.	–	Ref.	–	Ref.	–
1 or 2	4.286(0.510–36.009)	0.180	5.461(0.619–48.173)	0.126	0.909(0.200–4.133)	0.902	1.043(0.221–4.932)	0.958
≥3	4.611(0.581–36.616)	0.148	4.646(0.558–38.707)	0.156	1.387(0.340–5.656)	0.648	1.551(0.369–6.523)	0.550

BMI, body mass index.

Adjusted model: Adjusted for age, sex, BMI, alcohol use, smoking, vintage, hypertension, hyperlipidemia, heart disease, medicine use.

## 4 Discussion

Our study delineates the association between GNRI and depression in older dialysis patients with and without T2DM. We found that, among patients with T2DM, those with depression had significantly lower GNRI scores than their non-depressed counterparts; however, no such association was observed in patients without T2DM.

The detection rate of depressive symptoms in the PHQ-9 in our study was 35.1%, which is consistent with the results of other studies: King-Wing Ma et al. reported the prevalence of depressive symptoms in hemodialysis patients to be 22.8%–39.3% (7). However, several studies have shown a higher prevalence of depression in hemodialysis patients, as high as 85% (32). Heterogeneity across studies can be attributed to variations in patient characteristics, time since dialysis initiation, and the screening instruments employed.

Some studies suggest that serum hemoglobin (33, 34), albumin (34, 35), lipid levels (36), and heart disease (37) are associated with depression. In contrast, we did not observe an independent association between serum albumin and depressive symptoms. This discrepancy may partly reflect heterogeneity in patients’ metabolic status, body composition, physical-activity levels, environmental factors, and the severity spectrum of depression.

Older adults with diabetes are more likely to suffer from depression. Additionally, our investigation revealed that T2DM patients exhibited a significantly higher prevalence of depression compared to non-T2DM patients (*P*<0.05). This result is consistent with prior research (38, 39). Additionally, our previous study demonstrated notable nutritional disparities associated with depressive status (40). Malnutrition not only contributes to adverse health outcomes in individuals with diabetes but also serves as a risk factor for chronic kidney disease (CKD) (41, 42). Thus, in this study, we examined the link between the GNRI and depression in diabetic and non-diabetic hemodialysis patients as well as the nutritional status as assessed by the GNRI in the depressed and diabetic subgroups of the population receiving hemodialysis.

Potential mechanisms underlying the association between the GNRI and depression are outlined below. Depression is well-documented to be immune-modulated. Elevated levels of inflammatory markers, such as C-reactive protein (CRP), interleukin-6 (IL-6), interleukin-1 (IL-1), tumor necrosis factor-α (TNF-α), and soluble IL-2 receptor (43–45), are consistently associated with depression in cross-sectional study meta-analyses. Additionally, gene expression studies have discovered that depression is associated with an upregulation of inflammatory pathways (46–48). IL-6 and CRP represent the most extensively studied inflammatory biomarkers in this context. Both are easy to measure in serum and are related. Consequently, CRP and IL-6 are among the most frequently scrutinized serological indicators for depression.

Association between the GNRI and systemic inflammation have been documented in prior research (49, 50). A previous study has found that patients with lower GNRI scores had lower serum albumin concentrations and lean body mass and higher levels of inflammatory markers, including CRP, TNF-α, and IL-6 (49). These findings indicate that the GNRI—an accessible, validated nutritional screening tool—not only assesses nutritional status but also serves as a proxy for underlying inflammatory processes, particularly in patients with chronic kidney disease (CKD).

Diabetes is a chronic medical condition with a well-established bidirectional association with depression. The burden of diabetes management, along with diabetes-related complications and psychosocial stressors, such as stigma and the psychological impact of diagnosis, may trigger or exacerbate depressive disorders (51, 52). The onset of diabetes exacerbates concerns regarding protein–energy balance by accelerating the loss of muscle mass, strength (53), and serum albumin, while also adversely affecting overall nutritional intake. Hypoalbuminemia is associated with both acute and chronic inflammation, a key pathophysiological mechanism contributing to the development of long-term diabetic complications. Furthermore, prolonged chronic inflammation increases the risk of hypoalbuminemia, suggesting that as patients with diabetes accumulate chronic complications (54), systemic inflammatory activity is also heightened. Since GNRI is primarily influenced by serum albumin, height, and body weight objective factors, the index of GNRI decreases when these variables remain constant.

In the present study, the GNRI, when analyzed as a continuous variable, was significantly associated with depression in the diabetic population. Depressed patients exhibited lower GNRI scores compared to their non-depressed counterparts. Notably, the GNRI was not employed as a dichotomous indicator in this analysis, as such categorization may oversimplify the assessment of nutritional status and fail to capture subtle but clinically relevant changes that could influence patient prognosis. Furthermore, the observed associations may be influenced by sample selection and specific population characteristics. Therefore, further well-designed prospective cohort studies are warranted to validate the relationship between GNRI and depression in individuals with diabetes.

## 5 Limitations

While this study offers novel insights into the relationship between nutrition and depression in dialysis patients, both with and without diabetes, several limitations must be acknowledged. First, since this was a cross-sectional study, it was not possible to determine the existence of a causal link. Second, it is possible that the study sample cannot be applied to other populations because it was restricted to Chinese dialysis patients. Third, even after adjusting for a number of variables, we continue to take into account certain variables, such as residency status and marital status. Furthermore, the GNRI was only used as a continuous variable in this study and was not classified using the traditional methods. Consequently, large-scale multicenter studies with increased sample diversity are imperative to enhance statistical power and generalizability. Furthermore, longitudinal designs are essential for establishing temporal relationships, tracking GNRI-depression trajectories, and validating causality in this understudied population.

## 6 Conclusion

In conclusion, this study demonstrated the relationship between depression and the GNRI in older Chinese community dialysis patients, both with and without T2DM. Notably, a significant correlation was observed between depression and GNRI within the diabetic cohort, highlighting the potential impact of nutritional status on the onset of depression. These findings provide valuable insights for clinical practice and future research, potentially leading to improved mental health treatment for dialysis patients.

## Data Availability

The original contributions presented in the study are included in the article/supplementary material. Further inquiries can be directed to the corresponding author.
